# Karyotype, evolution and phylogenetic reconstruction in Micronycterinae bats with implications for the ancestral karyotype of Phyllostomidae

**DOI:** 10.1186/s12862-019-1421-4

**Published:** 2019-05-07

**Authors:** T. C. M. Benathar, C. Y. Nagamachi, L. R. R. Rodrigues, P. C. M. O’Brien, M. A. Ferguson-Smith, F. Yang, J. C. Pieczarka

**Affiliations:** 1 Laboratório de Citogenética, Centro de Estudos Avançados da Biodiversidade, Universidade Federal do Pará, Av. Perimetral, sn. Guamá, Belém, Pará 66077 Brasil; 2 PPGBionorte, Belém, State of Para Brazil; 3 Laboratório de Genética e Biodiversidade, ICED, Universidade Federal do Oeste do Pará, Belém, Brasil; 4 CNPq, Brasilia, Brazil; 5 Cambridge Resource Centre for Comparative Genomics, Department of Veterinary Medicine, University of Cambridge, Cambridge, UK; 6 Cytogenetics Facility, Welcome Trust Sanger Institute, Hinxton, UK

**Keywords:** Chromosome phylogeny, Interstitial telomeres, Cytotaxonomy, Genomic mapping

## Abstract

**Background:**

The Micronycterinae form a subfamily of leaf-nosed bats (Phyllostomidae) that contains the genera *Lampronycteris* Sanborn, 1949, and *Micronycteris* Gray, 1866 (*stricto* sensu), and is characterized by marked karyotypic variability and discrepancies in the phylogenetic relationships suggested by the molecular versus morphological data. In the present study, we investigated the chromosomal evolution of the Micronycterinae using classical cytogenetics and multidirectional chromosome painting with whole-chromosomes probes of *Phyllostomus hastatus* and *Carollia brevicauda*. Our goal was to perform comparative chromosome mapping between the genera of this subfamily and explore the potential for using chromosomal rearrangements as phylogenetic markers.

**Results:**

The Micronycterinae exhibit great inter- and intraspecific karyotype diversity, with large blocks of telomere-like sequences inserted within or adjacent to constitutive heterochromatin regions. The phylogenetic results generated from our chromosomal data revealed that the Micronycterinae hold a basal position in the phylogenetic tree of the Phyllostomidae. Molecular cytogenetic data confirmed that there is a low degree of karyotype similarity between *Lampronycteris* and *Micronycteris* specimens analyzed, indicating an absence of synapomorphic associations in Micronycterinae.

**Conclusions:**

We herein confirm that karyotypic variability is present in subfamily Micronycterinae. We further report intraspecific variation and describe a new cytotype in *M. megalotis*. The cytogenetic data show that this group typically has large blocks of interstitial telomeric sequences that do not appear to be correlated with chromosomal rearrangement events. Phylogenetic analysis using chromosome data recovered the basal position for Micronycterinae, but did not demonstrate that it is a monophyletic lineage, due to the absence of common chromosomal synapomorphy between the genera. These findings may be related to an increase in the rate of chromosomal evolution during the time period that separates *Lampronycteris* from *Micronycteris*.

**Electronic supplementary material:**

The online version of this article (10.1186/s12862-019-1421-4) contains supplementary material, which is available to authorized users.

## Background

The subfamily Micronycterinae of leaf-nosed bats (Phyllostomidae) was first recognized based on variations in the restriction site data [1]. The author included the genera *Glyphonycteris*, *Lampronycteris*, *Micronycteris* and *Trinycteris* in this subfamily. However, the understanding of relationships among the genera of this subfamily has undergone considerable changes in the years since 1996 [2–6].

Morphological analysis recognized six subgenera (*Glyphonycteris*, *Lampronycteris*, *Micronycteris*, *Neonycteris*, *Trinycteris* and *Xenoctenes*) within genus *Micronycteris* [2], whereas analyses of allozymes, karyotypes and morphologies [3] led researchers to propose five seemingly monophyletic subgenera (*Glyphonycteris*, *Lampronycteris*, *Micronycteris*, *Neonycteris* and *Trinycteris*). The subgenera described in the former analysis [2] were elevated to the status of genera in the latter [4]. Moreover, morphological data indicated that the genera were closely related, with *Macrotus*, *Micronycteris*, *Lampronycteris*, *Glyphonycteris*, *Trinycteris* and *Neonycteris* classified as belonging to tribe Micronycterini within the subfamily Phyllostominae [5]. *Micronycteris* and *Lampronycteris* have been proposed to represent a more restricted clade belonging to subfamily Micronycterinae, which was thought to represent a basal group within the Phyllostomidae that diverged after the Macrotinae and before the Desmodontinae [6]. This phylogenetic proposal differed markedly from previous hypotheses, as it implied that the primitive features present in *Micronycteris* (*lato* sensu) were not evidence of monophyly but rather represent symplesiomorphies [6]. More recently, some researchers have found significant statistical support for the monophyly of Micronycterinae [7–9] and confirmed their basal position. Others, however, have suggested that the morphological evidence indicates that the Micronycterinae diverged from the rest of the Phyllostomidae after the Desmodontinae [10].

Chromosomal studies in this subfamily has demonstrated that there is large variation among the known karyotypes, with diploid numbers ranging from 2n = 25 [11] to 2n = 40 and fundamental number ranging from FN = 30 to FN = 68 [3, 12–21]. Comparative analysis of G-banding in representatives of subfamily Micronycterinae revealed the presence of two chromosomal synapomorphies with respect to the karyotype of *Macrotus waterhousii*: a translocation (25/26–13) and a Robertsonian fusion (22/14) that are shared by *Lampronycteris brachyotis* and *Micronycteris minuta* [15]. At least 14 or 15 independent rearrangements distinguish the karyotypes of *M. minuta* and *M. megalotis* from that of *Macrotus waterhousii*, which is close to the hypothetical ancestral karyotype of the Phyllostomidae [22]. It is difficult to interpret the karyotypic data of genera *Lampronycteris* and *Micronycteris* because there is a high degree of karyotypic difference between these species and little cytogenetic information is available for this group; of 12 species described to date, karyotypic formulas are available for seven, most of which were generated using only conventional staining and G-banding [3].

Chromosomal data can contribute to our understanding of evolutionary relationships; as hereditary elements of the nuclear genome, chromosomes act as independent mutational units and thus meet important conditions for inclusion as characters in phylogenetic investigations [23]. The use of classical cytogenetics paired with multidirectional chromosome painting can enable researchers to make more detailed comparisons between the karyotypes of different taxa and to accurately identify rearrangements between species and/or genera. This may contribute significantly to the discussion of phylogenetic and cytotaxonomic issues [24–26].

Here, we investigate the karyotype evolution of subfamily Micronycterinae and estimate the magnitude of the chromosomal changes between the genera that compose this subfamily. We also integrated our genomic mapping results with published data from other species of subfamily Phyllostomidae in an effort to reconstruct the chromosomal phylogeny of the Micronycterinae.

## Results

### Karyotypic characterization and multidirectional chromosome painting in *Lampronycteris brachyotis*

**Fig. 1:**
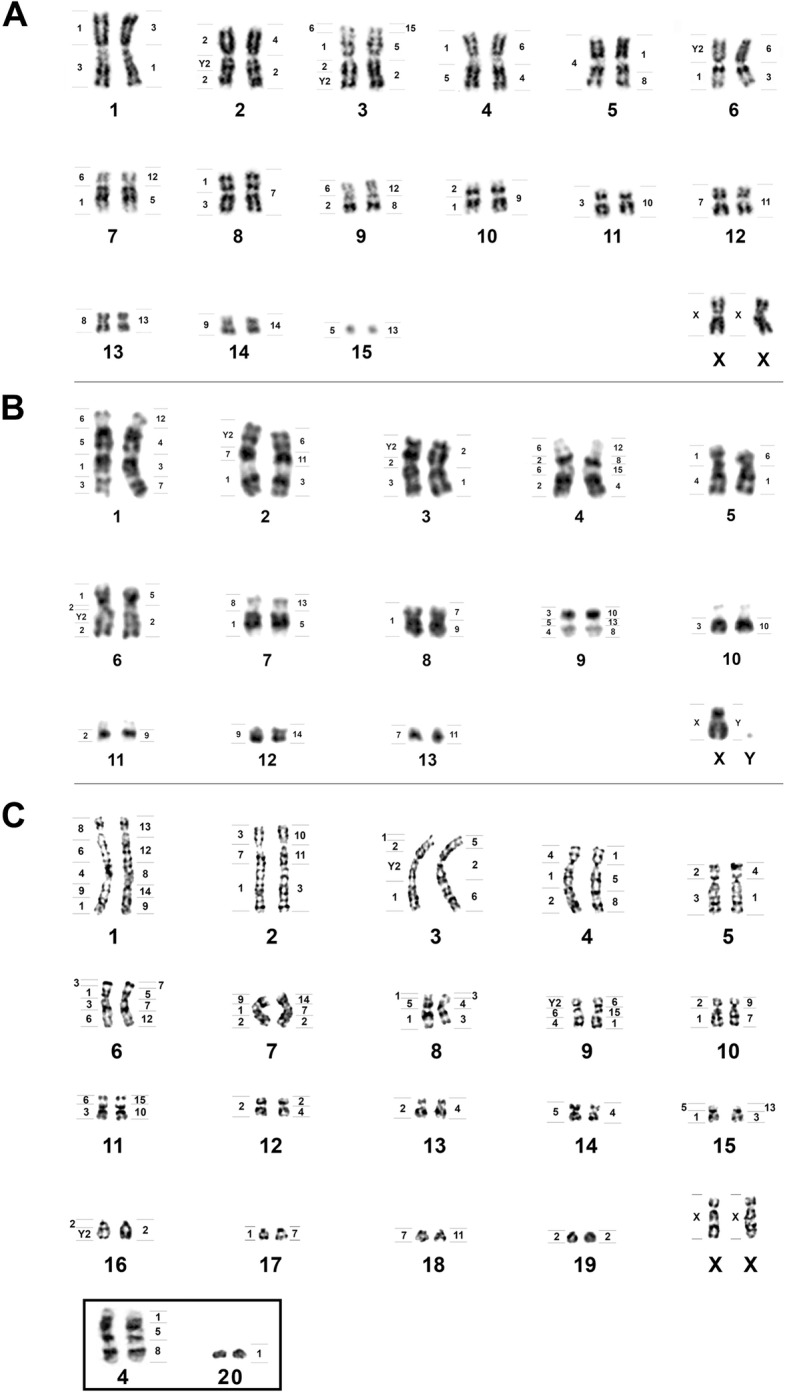
G-banded karyotypes showing mapping of the probes from CBR (left) and PHA (right). **a** *Lampronycteris brachyotis.* **b** *Micronycteris minuta.* **c** *Micronycteris megalotis*

**Fig. 2:**
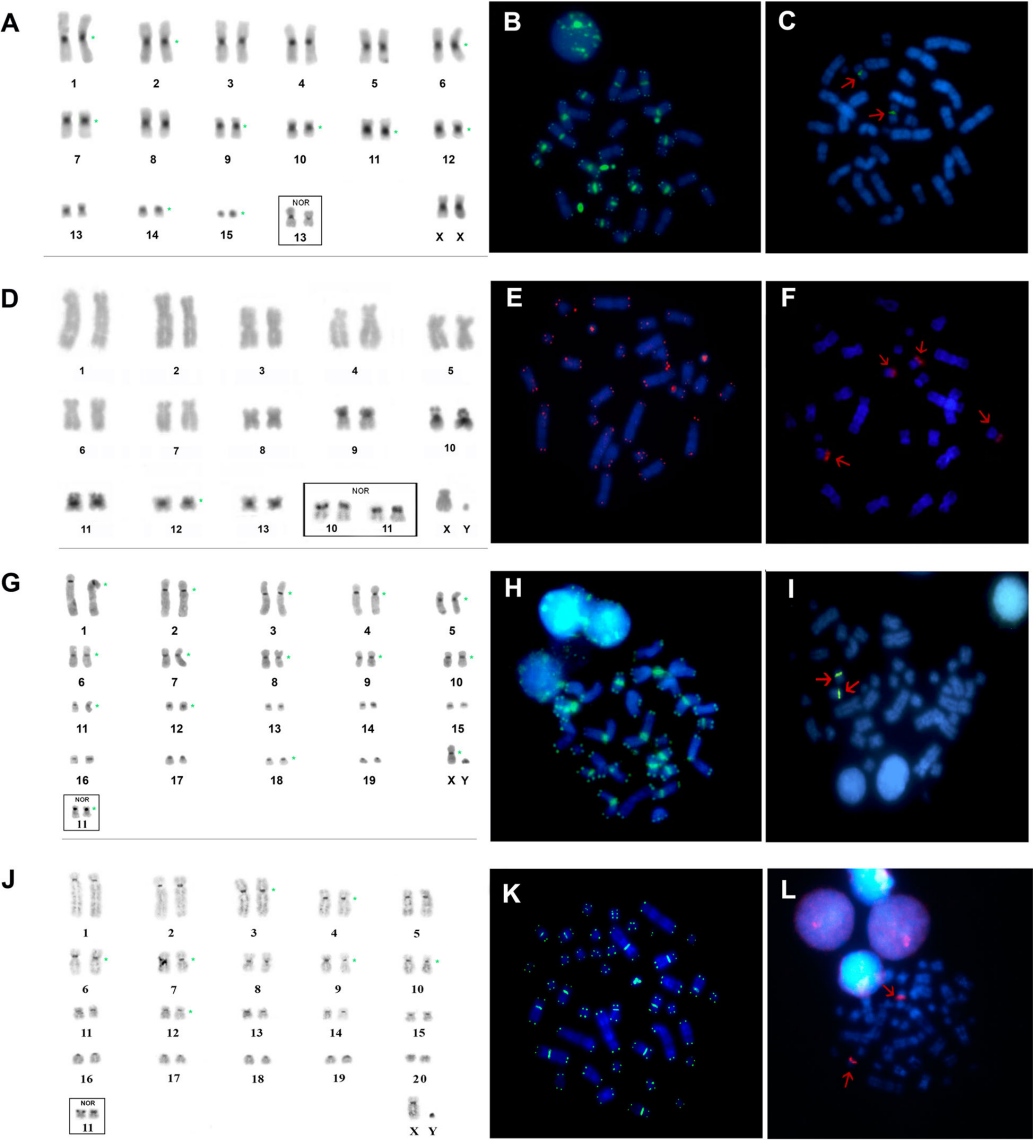
C-banding, NOR staining and FISH in the karyotypes of Micronycterinae. *Lampronycteris brachyotis*: **a** C-banding, **b** FISH with telomeric probes **c** FISH with rDNA 18S probe. *Micronycteris minuta*: **d** C-banding, **e** FISH with telomeric probes, **f** FISH with rDNA 18S probe. *Micronycteris megalotis* (2n = 40): **g** C-banding, **h** FISH with telomeric probes, **i** FISH with rDNA 18S probe. *Micronycteris megalotis* (new cytotype): **j** C-banding, **k** FISH with telomeric probes, **l** FISH with rDNA 18S probe. (NOR into the box)

The karyotype of *L. brachyotis* (LBR) is composed of 15 bi-armed autosomal pairs and has 2n = 32 and FN = 60. The X is medium-sized and metacentric (Fig. 1a). Constitutive heterochromatin is found in the centromeric regions of all chromosome pairs (Fig. 2a). A Nucleolar Organizer Region (NOR) is present in the pericentromeric region of pair 13 (Fig. 2a, box). Interstitial telomeric sequences (ITSs) are present at the distal portion of each chromosome, as well as in the centromeric regions of pairs 1, 2, 6, 7, 9–12, 14 and 15 (Fig. 2a). 18S rDNA sites are seen in the pericentromeric region of pair 13 (Fig. 2a). FISH with whole-chromosome probes from CBR and PHA detected 29 and 26 homologous segments, respectively (Fig. 1a).

### Karyotypic characterization and multidirectional chromosome painting in *Micronycteris minuta* and *M. homezi*

*Micronycteris minuta* (MMI) and *M. homezi* (MHO) have similar karyotypes with 2n = 28/FN = 52 and 13 bi-armed autosomal pairs. The X is medium-size and submetacentric, and the Y is small and acrocentric (Fig. 1b). Constitutive heterochromatin is found only in the centromeric regions of the small chromosome pairs, 9–13 (Fig. 2d). NORs are found in the proximal regions of the short arms of chromosome pairs 10 and 11 (Fig. 2d, box). ITSs are found at the distal portion of each chromosome and in the pericentromeric region of pair 12 (Fig. 2f). Hybridization of the 18S rDNA probe is seen in the proximal regions of the short arms of pairs 10 and 11 (Fig. 2e). Multidirectional chromosome painting using the CBR and PHA probes identified 31 and 29 homologous segments, respectively, in MMI (Fig. 1b). Due to the karyotypic similarities between MMI and MHO, we inferred that they would share the same syntenic groups and thus the chromosome painting data of MMI can be extrapolated to MHO.

### Karyotypic characterization and multidirectional chromosome painting in *Micronycteris megalotis* and *M. microtis*

The karyotypes of *Micronycteris megalotis* (MME) and *M. microtis* (MMC) obtained from the states of Amazonas and Pará are quite similar, exhibiting 2n = 40/FN = 68 with 15 bi-armed autosomal pairs and four acrocentric pairs. The X is medium-size and submetacentric, while the Y is small and acrocentric (Fig. 1c). Constitutive heterochromatin is found in the centromeric region of all chromosomal pairs (Fig. 2g). The NOR is located in the proximal region of the short arm of pair 11 (Fig. 2g, box). ITSs are seen in the distal portions of all chromosomes and in the centromeric regions of pairs 1–12, pair 18 and the sex chromosomes (Fig. 2h). Labeling of 18S rDNA sites is found in the proximal region of the short arm of pair 11 (Fig. 2i).

*Micronycteris megalotis* from the Municipality of Laranjal do Jari (AP) presented a new cytotype of 2n = 42/FN = 70, with 15 bi-armed autosomal pairs and five acrocentric pairs. The X is medium-size and submetacentric, and the Y is small and acrocentric. This chromosomal formula differs from the reported karyotype of 2n = 40/FN = 68 by an additional a pair of acrocentric chromosomes (pair 20; Fig. 1c, box). The C-banding pattern showed that there is constitutive heterochromatin in the centromeric regions of all chromosomal pairs (Fig. 2j). The NOR is located in the proximal region of the short arm of pair 11 (Fig. 2j). ITSs were found at the distal portions of all chromosomes and in the centromeric region of pairs 3, 4, 6, 7, 9, 10 and 12 (Fig. 2k). FISH with 18S rDNA probes showed hybridization in the proximal region of the short arm of pair 11 (Fig. 2l).

**Fig. 3:**
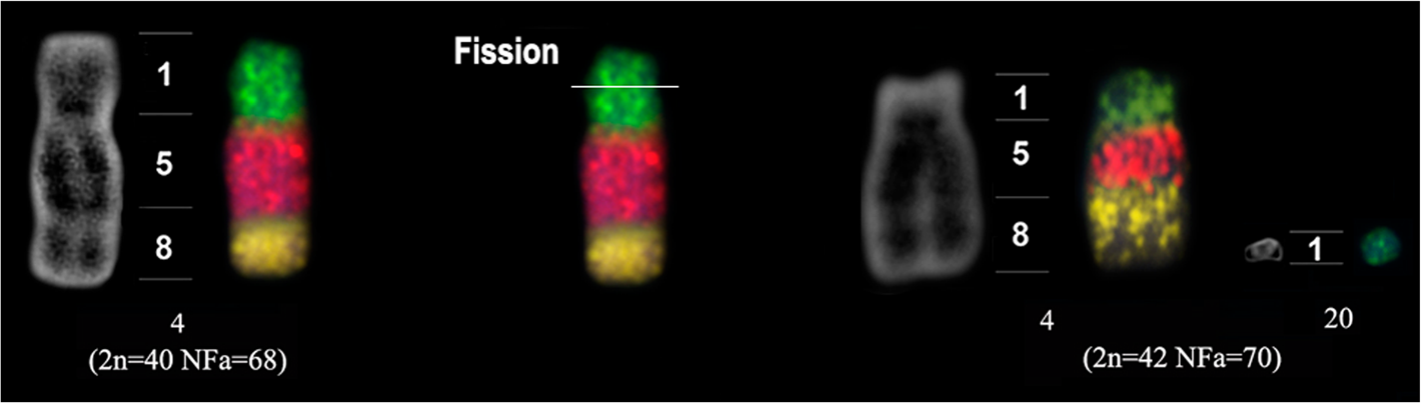
Chromosome painting showing the fission involved in the origin of pair 20 of *Micronycteris megalotis* (new cytotype)

Chromosome painting using the CBR and PHA probes revealed the presence of 45 and 43 homologous segments, respectively, in MME with 2n = 40 (Fig. 1c). Due to the karyotypic similarities between MME and MMC, we inferred that they would share the same syntenic groups and therefore the chromosome painting data obtained from MME could be extrapolated to MMC. As for MME (new cytotype), only the associations corresponding to MME chromosome 4 (2n = 40) (PHA 1, 5 and 8) were hybridized to demonstrate that a fission event involving the short arm of chromosome 4 generated pair 20 in MME (new cytotype) (Fig. 3).

### Phylogenetic analysis using chromosomal rearrangements as characters

**Fig. 4:**
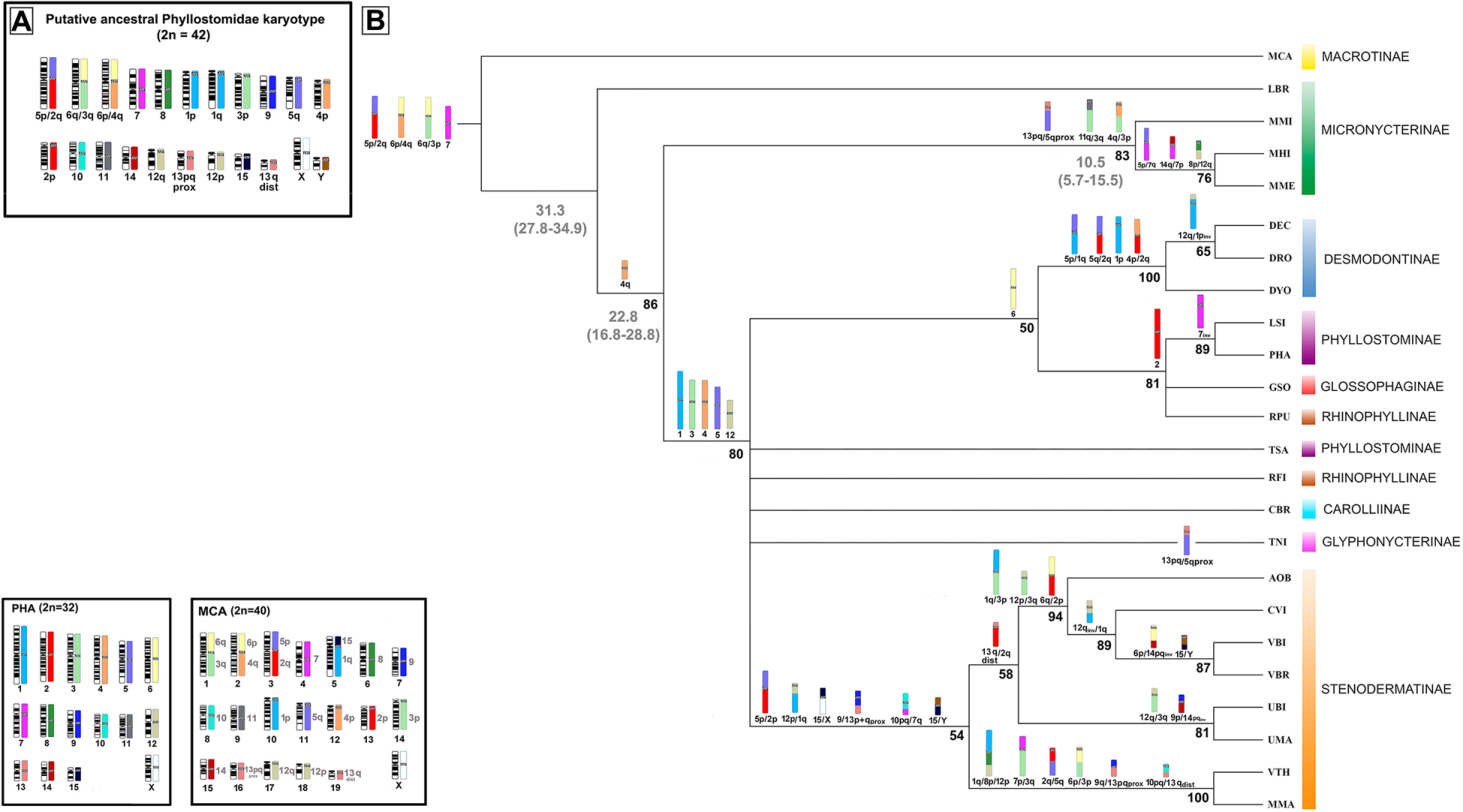
Analysis of the chromosome characters. **a** Ideogram of the putative ancestral karyotype of Phyllostomidae. **b** Maximum parsimony tree obtained by the software PAUP. Boxes below show the ideogram of the *Phyllostomus hastatus* karyotype ​​from which the PHA whole chromosome probes were made and the ideogram of *Macrotus californicus* karyotype with the mapping of PHA whole chromosome probes. Abbreviations of species names are described in Table 1. Symbols: “p” = short arm; “q” = long arm; “/” = Syntenic groups physically linked; “Inv” = inversion; Bold numbers (over the branches) are the bootstrap values for 1000 replicates

**Fig. 5:**
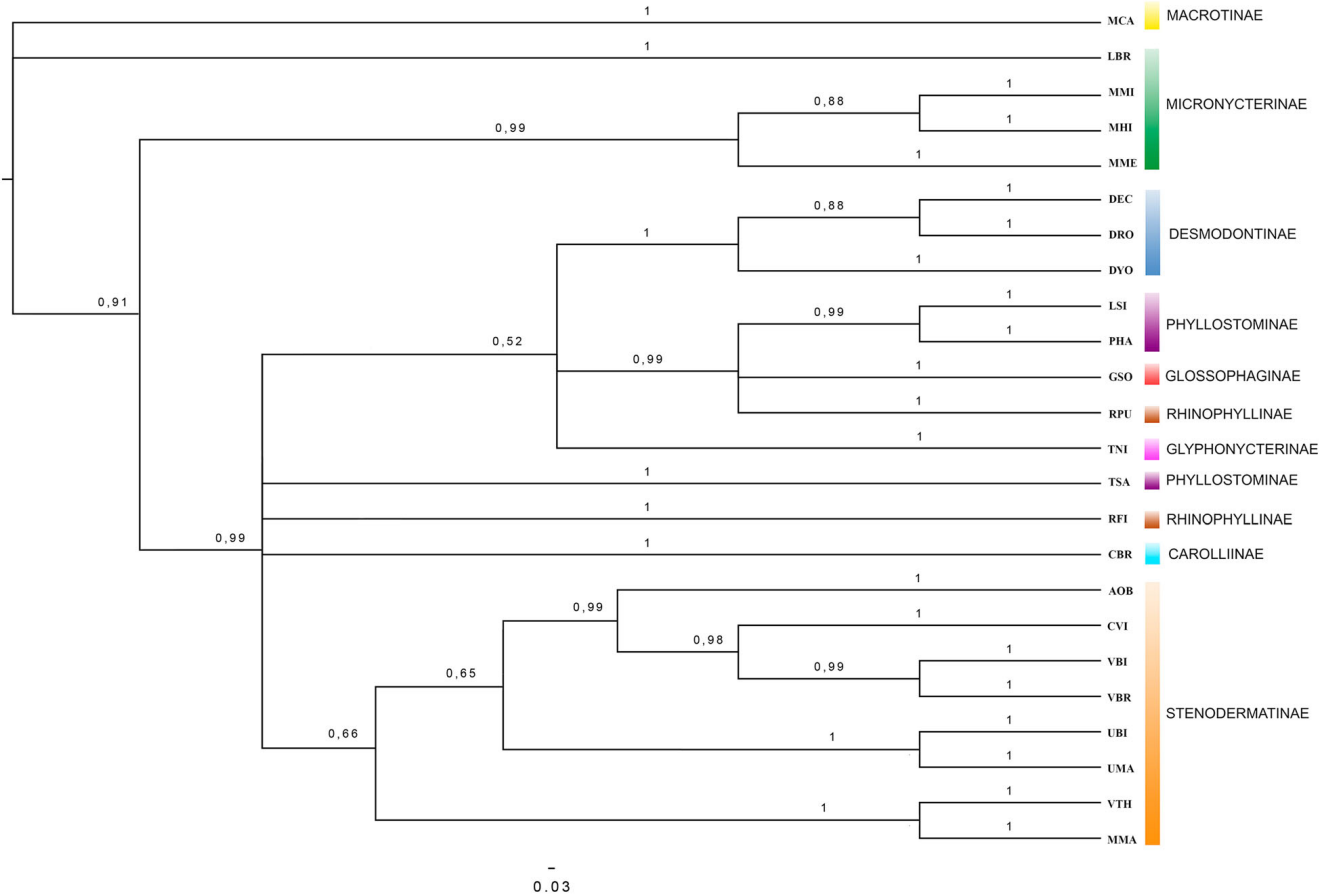
Phylogenetic reconstruction using Bayesian analysis. Numbers close to nodes: estimated a posteriori probabilities

Fourteen equally parsimonious trees (L = 104, CI = 0.56, RI = 0.72 excluding non-informative characters) were obtained by Maximum Parsimony (MP) analysis (Fig. 4b). Further analysis was performed using the Bayesian method (later probabilities are shown in the branches). Figure 5 shows the consensus tree. Both topologies are weakly supported by bootstrap and posterior probability values for the clade that groups subfamilies Desmodontinae, Phyllostomidae, Glossophaginae and Rhinophyllinae, as well as for two clades within Stenodermatinae. In general terms, the recovered phylogenetic pattern is consistent with the previously published reconstructions based on molecular and morphological data, with the exception of Micronycterinae, which is paraphyletic in our analysis (but see Discussion).

## Discussion

### Karyotypic diversity in Micronycterinae

The karyotypic data compared herein agree with those of previous studies [11, 12, 14, 19] for LBR and MMC, but are discordant in some cases for MMI and MME.

Our morphological analysis of chromosomes from *Micronycteris minuta* from different sites found that the X chromosome is submetacentric, which does not agree with an earlier report that described the X as metacentric [15]. Comparison of our G-banding and chromosome-painting data with the published results indicates that the X chromosomes identified in the prior report [15] actually correspond to pair 8 in our analysis. This likely reflects that the authors of the previous paper analyzed only one female.

For specimens of MME from the states of Pará, Amazonas and Amapá, our karyotypic results are in agreement with the data described in previous studies, presenting 2n = 40 and FN = 68 [15, 16, 19]. However, the specimen from the municipality of Laranjal do Jari had 2n = 42/FN = 70. This provides evidence for intraspecific variation in specimens distributed to the state of Amapá and stands as the largest FN described to date for family Phyllostomidae. Specimens have karyotypes highly similar in their banding patterns and localization of the ribosomal sites, but differed in their distribution of telomeric sequences (Fig. 2h and k) and also by the presence of an additional acrocentric pair (pair 20), that arose from a fission of the short arm of pair 4, as indicated by chromosome painting (see Fig. 3). A similar result was reported for *M. hirsuta* (MHI), with the presence of different cytotypes described even though the taxon was reported to be monotypic [21]. These findings demonstrate that genus *Micronycteris* has a large degree of intraspecific karyotypic diversity, reinforcing the need to investigate species that have not yet been subject to cytogenetic analysis, such as *M. buriri*, *M. brosseti*, *M. matses* and *M. yatesi*.

All of the ITSs found in the species analyzed herein matched with the results of our constitutive heterochromatin (CH) banding. In MME (2n = 40), the ITS of pair 11 co-locates with CH and the NOR (Fig. 2g and h). The same pattern of ITS distribution was found in MHI, which is consistent with a previous report [21]. We found no correlation between ITSs and chromosomal rearrangements in Micronycterinae, as the ITS signals were not located at regions of syntenic association where fusions have occurred, and the signals were found on conserved chromosomes 9, 10, 11 and 14 relative to PHA. Our analysis suggests that the ITSs of the studied species appear to be strongly associated with a specific class of ITSs, called heterochromatic ITSs [41], which comprise large blocks of telomere-like sequences and are usually located within or at the edge of constitutive heterochromatin. This type of ITS has been described in several vertebrate species [42–44]. Even though ITSs do not act as functional telomeres and their biological functions have not yet been elucidated [41, 45–47], their distribution patterns suggest that these sequences may play significant roles in genomic instability and chromosomal evolution.

### Cytotaxonomy of *Micronycteris homezi* and *M. microtis*

Although the taxonomic status of MHO and MMC is still up for debate due to the lack/overlap of diagnostic characters, which complicate their differentiation [48, 49], MHO and MMC are recognized as valid species [3, 4]. A previous report proposed that there is synonymy between MHO and MMI [49], with the latter acting as the senior synonym. The cytogenetic data presented in the present work demonstrate that the karyotypes of MHO and MMC are very similar, with no divergence visualized with the utilized techniques. We were thus unable to differentiate these taxa by karyotype. This corroborates the difficulty of assigning MHO and MMC and suggests that these taxa will warrant further taxonomic review.

### Genomic organization and evolution of Micronycterinae bats

Per a review of the molecular and morphological data [50], the existing studies agree that subfamily Micronycterinae consists of genera *Lampronycteris* and *Micronycteris* and is a monophyletic lineage. However, the studies do not agree on its phylogenetic position; the molecular data strongly support a basal position [6–9], while the morphological data place this lineage within the clade of subfamily Phyllostominae [10].

Despite of cladistic and Bayesian analysis described herein recovered an organization that differed from those of the previous reports due to the absence of synapomorphic associations between *Lampronycteris* and *Micronycteris*, this does not necessarily means that Micronycterinae is not a monophyletic lineage, since the data point to a common characteristic, at least in LBR and MMI (PHA 8q/12q). This character could represent a synapomorphic association for Micronycterinae in the past and that was later modified by a WART in MME and MHI, leading to the formation of chromosomes 1 and 4 in MME and 5 and 6 in MHI. Furthermore chromosome 4 of MME and 6 of MHI were modified by the addition of one more segment. Our findings agree with the molecular data regarding these phylogenetic positions [6–9], with Micronycterinae representing a basal group within Phyllostomidae that diverged after Macrotinae and before Desmodontinae.

The chromosome painting data confirmed that associations with PHA 12p/5q and 15/5p define LBR, whereas those with PHA 11q/3q and 4q/3p define *Micronycteris* and that there is a low degree of karyotype similarity between LBR and the examples of Micronycteris analyzed, based on the retention of two synergistic associations: PHA 8q/12q (CI = 0.50) with MMI; and PHA 5p/2q (CI = 0.33) with MMI and MME, which is a synthetic ancestral association present in MCA. LBR also retains two more ancestral syntenic associations, 6p/4q (CI = 0.50) and 6q/3p (CI = 1), supporting its basal position. In this context, our data refute a previous proposition regarding the synapomorphies present in *Micronycteris* (sensu *lato*), wherein the authors posited that a certain translocation (25/26–13), based on the karyotype of *Macrotus waterhousii* (MWA) is shared between LBR and MMI [15]. This association would be homologous to PHA 13q prox/5q (CI = 0.33), which is present only in MMI, MHI and *Trinycteris nicefori* (Glyphonycterinae). The Robertsonian fusion, MWA 22/14 (PHA 5p/2q), would thus be the only shared syntenic association between LBR and MMI [15]. However, it would not be exclusive to Micronycterinae, as indicated by our data.

Although our findings do not corroborate the monophyly of Micronycterinae, we believe that this does not justify reorganization of genus *Lampronycteris* into another subfamily. We conclude that in cases like this, phylogenetic systematics needs to be pluralistic. This will improve our understanding of complex lineages (such as Micronycterinae) by allowing researchers to integrate evidence accumulated from different sources, including morphological, molecular, ecological, chromosomal and chromosome-speciation models [7, 51–53].

In an effort to elucidate what might have happened in the radiation of lineages whose karyotypes differ to the point that no chromosomal synapomorphy can be traced, we performed a brief integration of our data with the molecular data [7]. With respect to the divergence of *L. brachyotis*, which began around 22.8 MYA, we observed that the present lineage shows a marked karyotypic conservation characterized by the presence of 11 pairs of syntenic chromosomes found in the ancestor of Phyllostomidae and only eight rearrangements (two fissions and six fusions), suggesting a reduced rate of chromosome evolution. In contrast, *Micronycteris*, which has a more recent origin, beginning around 10.5 MYA, shows extensive chromosomal diversification involving the fixation of different rearrangements that occurred over a relatively short time when compared to LBR. Among the analyzed species, we observed the following differences with respect to the ancestor of Phyllostomidae: for MMI, eight fissions, 15 fusions and two inversions; for MHI, nine fissions, 20 fusions and two inversions; and for MME, 16 fissions, 23 fusions and three inversions. Thus, there is evidence for extensive karyotypic reorganization. We suggest that the absence of chromosomal synapomorphy in the Micronycterinae may be associated with an increase in the chromosomal change rate in relation to the period of time that separates successive events of divergence between the two strains (~ 12.3 MYA). Although the basis for the high rates of genomic remodeling observed herein is not yet clearly understood, some authors have posited that factors such as the population structure and the content of repetitive DNA may contribute to modulating the rate of karyotypic evolution [52, 54, 55].

### Chromosomal signatures: implications for the ancestral karyotype of the Phyllostomidae (AKP)

Our chromosomal mapping data of Micronycterinae together with data from the literature [37, 38, 56] enabled us to deduce a new ancestral karyotype for Phyllostomidae (AKP) based on our inference of chromosomal signatures and whole-chromosome syntenies.

The first ancestral karyotype proposed for the family [15] was 2n = 46/FN = 60 (with 16 bi-armed autosomal pairs, 14 acrocentric pairs and the sex chromosomes); of the relevant species, only *Macrotus waterhousii* retained this ancestral state [15, 57, 59]. The ancestral karyotype of Phyllostomidae inferred from a previous chromosome-painting analysis [38] was 2n = 42/FN = 60, with 11 bi-armed chromosome pairs (including the X chromosome) and 10 acrocentric pairs. In the present study, we observed the same diploid and fundamental number as the latter study. However, our comparisons of syntenic associations and whole-chromosome syntenies suggested that our AKP would differ from the previously proposed ancestral karyotype, as follows: (1) PHA3 and PHA6 in the ancestor proposed herein would be involved in the syntenic associations PHA 6q/3q and 6p/4q, whereas 3p would be in the free acrocentric form; (2) the PHA 5p and 2p segments would be involved in the PHA 5p/2p association; and (3) the 12p/1q association would be dissociated in free forms. All other chromosomes are consistent with the previous report [38].

We conclude that our proposed AKP is the most likely candidate for the primitive condition of Phyllostomidae (Fig. 4a). Essentially, its karyotype would be very close to that of *Macrotus californicus*. This conclusion is supported by the presence of eight whole-chromosomes syntenies in most of the subfamilies of Phyllostomidae, namely PHA 8–11, PHA 13p + q prox (CBR 8), PHA 13q dist (CBR 5), PHA 14 and PHA 15. Of the proposed ancestral karyotypic associations, PHA 6q/3q, 6p/4q and 5p/2q are all shared among the members of subfamily Micronycterinae, which represents a basal lineage for Phyllostomidae. Therefore, it is likely that these whole-chromosome syntenies and syntenic associations are plesiomorphic characters present in the different karyotypes of the studied Phyllostomidae.

### Use of chromosomal data to infer phylogenetic relationships among the Phyllostomidae

Chromosomal rearrangements are units of independent mutation, and thus are relevant as phylogenetic characters [23]. Given that the use of reciprocal chromosome painting between species can yield a relatively precise definition of structural rearrangements [24, 33, 35, 58], molecular cytogenetics can allow researchers to evaluate homologies between distantly related taxa. This has opened new opportunities for determining chromosomal relationships at higher taxonomic levels in mammals [25]. In such work, the members of family Phyllostomidae have been well studied due to their high levels of karyotypic variation [52].

Since chromosome painting is available for different subfamilies of Phyllostomidae [21, 33, 35–38], here we used chromosomal rearrangements as phylogenetic characters to investigate evolutionary relationships at various taxonomic levels within this family. We also newly report the chromosomal mapping of Micronycterinae, which is one of the most basic clades of this family. Our reconstruction was based on data from nine of the 10 subfamilies of Phyllostomidae, and parsimony (Fig. 4b) and Bayesian inference (Fig. 5) analyses yielded trees that were very similar to each other but discordant relative to the more robust molecular phylogenetic trees [6, 9] and a chromosome analysis-based tree [37].

Our comparisons yielded interesting results. For example, most of the derived associations were phylogenetically informative in the terminal portions of the branches, and the findings were consistent with the subfamily grouping (Desmodontinae and Stenodermatinae) and agreed with reconstructions based on molecular [6, 9] and morphological [5, 10] data. This indicates that chromosomal rearrangements and synteny contain high degrees of phylogenetic information. However, few associations could be identified to support the deeper divergences between many of the studied subfamilies, such as Phyllostominae, Glossophaginae, Rhinophyllinae and Glyphonycterinae. Representative species of these clades show many similar karyotypes, retaining most of the Robertsonian fusions that gave rise to the karyotype of PHA. Thus, the characters representing these fusions had a very low consistency index (IC < = 0.5). The presence of a high degree of whole-chromosome synteny at the basis of each subfamily line suggests that there was conservation prior to the radiation of the monophyletic subfamilies [35]. We interpret this finding as indicating that chromosome structures are much more complex than previously recognized for Phyllostomidae and that their chromosomal data, like those obtained using other markers, differ in their applicability depending on the taxonomic level under investigation.

Parsimony and Bayesian our analysis revealed that clades Phyllostominae and Rhinophyllinae are paraphyletic. This can be easily explained by the absence of a phylogenetic signal in the karyotypes of TSA and RFI, which do not share any syntenic association with other representatives of the clade. Both taxa share only syntenies of ancestral whole chromosomes and some Robertsonian fusions that gave rise to the current karyotype of PHA [37, 60].

## Conclusions

We herein confirm that a large karyotypic variability is present in Micronycterinae and newly report that there is intraspecific variation in *M. megalotis*, as shown by the description of a new cytotype (2n = 42) that offers the largest diploid number for Micronycterinae and the largest fundamental number (FN = 70) reported to date for Phyllostomidae. Large blocks of ITSs that coincide with constitutive heterochromatin, but show no obvious correlation with chromosomal rearrangements, characterize this subfamily. Phylogenetic analysis performed using the chromosomal data recovered the basal position for Micronycterinae but did not demonstrate that it was a monophyletic line. We did not find a syntenic association between *Lampronycteris* and *Micronycteris*, and instead observed distinct cytogenetic signatures in each lineage. The absence of chromosomal synapomorphy in Micronycterinae may be associated with an increase in the rate of chromosomal change in relation to the time period that separates successive events of divergence between *Lampronycteris* and *Micronycteris.*

## Methods

### Specimens examined

Thirty-four specimens were captured (two *Lampronycteris brachyotis*, 10 *Micronycteris megalotis*, three *M. microtis*, one *M. homezi* and 18 *M. minuta*) during expeditions to wildlife inventories in the states of Amapá, Amazonas, Pará and Mato Grosso (Additional file 1). Voucher specimens were deposited in the Collection of Mammals at the Museu Paraense Emilio Goeldi (MPEG), the Collection of Mammals of the Museum at the Federal University of Mato Grosso (CMUFMT), the Museum of Zoology of the Federal University of the West of Pará and the Collection of Mammals of the Research Institute Scientific and Technological Department of the State of Amapá (IEPA) (Additional file 1). Euthanasia of the specimens was performed according to Resolution 1000/2012 from the Brazilian Federal Council of Veterinary Medicine, by using intraperitoneal injection of barbiturate (Pentobarbital, 120 mg/kg) after local anesthetic (lidocaine used topically).

### Chromosomal preparations, cell culture and chromosome banding

Chromosomal preparations were obtained from bone marrow [27]. Primary culture of fibroblasts [28], G-banding, C-banding and Ag-NOR staining ([29–31], respectively) were performed as previously described.

### Fluorescence in situ hybridization (FISH)

FISH with telomeric probes was performed using All Human Telomere Probes (Oncor) according to the manufacturer’s protocol. FISH with 18S rDNA was performed using probes from *Prochilodus argenteus* [32]. Both were labeled with biotin by nick translation. Multidirectional chromosome painting was performed using whole-chromosome probes from *Phyllostomus hastatus* (PHA) and *Carollia brevicauda* (CBR), which were generated by flow cytometry [33]; the chromosome painting was performed as described in the literature [33, 34]. Briefly, the slides were pre-treated with acetic acid (50%) and methanol (100%), incubated in pepsin solution, dehydrated in an ethanol series (70, 90 and 100%), dried at room temperature and aged in an incubator at 65 °C for 1 h. The chromosomal DNA was denatured with 70% formamide diluted in 2xSSC at 62 °C for 50 s. The slides were immersed immediately in 70% ice-cold ethanol for 4 min and dehydrated with the above-described ethanol series. After hybridization for 72 h at 37 °C in a hybridization solution (14 μl of solution containing 50% formamide, 2x SSC, 10% dextran sulfate, 5 μg of salmon sperm DNA, 2 μg of mouse Cot-1 DNA and 1 μl of labeled PCR product) the slides were washed at 40 °C (2 × 50% formamide; 1x 2xSSC; 1x 4xSSC/Tween), and the metaphase chromosomes were stained with DAPI (4′, 6-diamidino-2-phenylindole).

### Image capture and data processing

Images were obtained using a Nikon Eclipse fluorescence microscope (H550S) equipped with a DS-Q1Mc (Nikon) camera. The NIS Elements software was used for camera control and digital image acquisition. For assignment of hybridization signals, specific chromosomes or chromosomal segments were identified using inverted DAPI-banding, which resembles the G-banding pattern. For image processing, the Adobe Photoshop CS6 software was used.

### Analysis of published data

Chromosome painting data were retrieved from the literature for 20 species of Phyllostomidae belonging to subfamilies Macrotinae [35], Desmodontinae [36], Phyllostominae [33], Glossophaginae [37], Rhinophyllinae [37], Carolliinae [33], Glyphonycterinae [37] and Stenodermatinae [38, 61]. These data were revised and analyzed (see Table 1 for the list of species). A total of 63 characters were identified and coded based on the presence/absence of syntenic associations with *P. hastatus* at both the ingroup and outgroup levels, as shown in the data matrix (Additional file 2).

**Table 1:** Literature data used in the analysis of chromosome mapping

Subfamily	Species	Abbreviation	2n	FN	Probes	Reference
Macrotinae	*Macrotus californicus*	MCA	40	60	MCA	[35]
Micronycterinae	*Lampronycteris brachyotis*	LBR	32	60	PHA/CBR	Present study
	*Micronycteris minuta*	MMI	28	50	PHA/CBR	
	*Micronycteris megalotis*	MME	40–42	68–70	PHA/CBR	
	*Micronycteris hirsuta*	MHI	25–26	32	PHA/CBR	[11]
Desmodontinae	*Diphylla ecaudata*	DEC	32	60	PHA/CBR	[36]
	*Diaemus youngi*	DYO	32	60	PHA/CBR	
	*Desmodus rotundus*	DRO	28	52	PHA/CBR	
Phyllostominae	*Phyllotomus hastatus*	PHA	32	58	CBR	[33]
	*Tonatia saurophila*	TSA	16	20	PHA/CBR	[60]
	*Lophostoma silvícola*	LSI	34	60	PHA/CBR	[60]
Glossophaginae	*Glossophaga soricina*	GSO	32	60	PHA, MCA	[35, 37]
Carollinae	*Carollia brevicauda*	CBR	20–21	36	PHA	[33]
Glyphonycterinae	*Trinycteris nicefori*	TNI	28	52	PHA/CBR	[37]
Rhinophyllinae	*Rhinophylla pumilio*	RPU	34	62	PHA/CBR	
	*Rhinophylla aff. Fischeraea*	RFI	38	68	PHA/CBR	
Stenodermatinae	*Artibeus obscurus*	AOB	30–31	56	PHA/CBR	[38]
	*Uroderma magnirostrum*	UMA	36	62	PHA/CBR	
	*Uroderma bilobatum*	UBI	42	50	PHA/CBR	
	*Chiroderma villosum*	CVI	26	48	PHA/CBR	[61]
	*Mesophylla macconnelli*	MMA	21–22	18	PHA/CBR	
	*Vampyressa thyone,*	VTH	23–24	20	PHA/CBR	
	*Vampyriscus bidens*	VBI	26	48	PHA/CBR	
	*Vampyriscus brocki*	VBR	24	44	PHA/CBR	

### Phylogenetic analysis

Phylogenetic analysis was performed using a matrix of binary data that represented the presence or absence of discrete characters. Chromosomal rearrangements were taken as representing the character, and the states of character were classified as plesiomorphic or apomorphic. The chromosomal complement of *Phyllostomus hastatus* (PHA) was used as a reference to define syntenic associations, since this species contains most of the ancestral segments of the Phyllostomidae family [15, 38]. *Macrotus californicus* (MCA) was used as outgroup because it holds the basal position in most of the published molecular phylogenies, and because it represents a monophyletic subfamily with well-supported molecular and chromosomal synapomorphies [9, 35]. A priori polarization of the characters was performed to define the direction in which the character states transformed.

The dataset was submitted to a maximum parsimony analysis using PAUP 4.0b10 [39] with the exhaustive search option. The robustness of the trees was explored through the consistency index (CI) and the retention indexes (RI), and the relative stability of the nodes was evaluated by bootstrap estimates based on 1000 replicates. A Bayesian inference (BI) was performed using version 3.2 of MrBayes [40]. The first 2 million generations were discarded as burn-in and the remaining trees were used to construct a majority-rule consensus tree and estimate the support values for each node.

### Definition of the ancestral karyotype

We integrated the chromosome-painting data obtained in the present work with those from literature [38, 57, 58, 61] to determine the syntenic associations present in the karyotype of the common ancestor of Phyllostomidae, as well as to determine syntenic associations or chromosomal blocks that were present in the basal nodule of each subfamily, using *M. californicus* as an outgroup. Chromosome morphology and banding patterns were evaluated to reveal possible inversions.

## Additional files

Additional file 1: Table of specimens analyzed. Species, Number of individuals/sex, locality, state of origin (SO), diploid number (2n), fundamental number (FN) and deposit of specimen vouchers analyzed in the present study. Museu Paraense Emilio Goeldi (MPEG); Collection of Mammals Museum of the Federal University of Mato Grosso (CMUFMT); Museum of Zoology, Federal University of Western Pará (MZUFOPA); Institute of Scientific and Technological Research of the State of Amapá (IEPA). (DOCX 22 kb)

Additional file 2: Basic data matrix of chromosomal rearrangements. The numbers of chromosomes are equivalent to those from PHA. Symbols: “p” = short arm; “q” = long arm; “/” = Synthetic groups physically linked; “Inv” = inversion; “M” = metacentric, “sm” = submetacentric; “A” = acrocentric. (DOCX 47 kb)

## Abbreviations

2n: Diploid number
Ag-NOR: Silver staining of Nucleolar Organizer Region
AKP: Ancestral karyotype of the Phyllostomidae
AP: Amapa state
BI: Bayesian inference
CBR: *Carollia brevicauda*
CH: Constitutive heterochromatin
CI: Consistency index
CMUFMT: Collection of Mammals at the Museum in the Federal University of Mato Grosso
DAPI: 4′, 6-diamidino-2-phenylindole
FISH: Fluorescence in situ hybridization
FN: Fundamental number
IEPA: Instituto de Pesquisa Científicas e Tecnológicas do Estado do Amapá
ITS: Interstitial telomeric sequence
LBR: *Lampronycteris brachyotis*
MCA: *Macrotus californicus*
MHI: *Micronycteris hirsuta*
MHO: *Micronycteris homezi*
MMC: *Micronycteris microtis*
MME: *Micronycteris megalotis*
MMI: *Micronycteris minuta*
MP: Maximum Parsimony
MPEG: Museu Paraense Emilio Goeldi
MWA: *Macrotus waterhousii*
MYA: Millions of years ago
NOR: Nucleolar Organizer Region
PHA: *Phyllostomus hastatus*
RFI: *Rhinophylla aff. fischerae*
RI: Retention index
SSC: Saline Sodium Citrate
TSA: *Tonatia saurophila*
