# Supplementary material for: Karyotype, evolution and phylogenetic reconstruction in Micronycterinae bats with implications for the ancestral karyotype of Phyllostomidae
Source: BMC Evol Biol. 2019 May 7;19:98. doi: 10.1186/s12862-019-1421-4 (PMC6505122; doi:10.1186/s12862-019-1421-4)
Supplement: Supplementary file 1 — Table of specimens analyzed. Species, Number of individuals/sex, locality, state of origin (SO), diploid number (2n), fundamental number (FN) and deposit of specimen vouchers analyzed in the present study. Museu Paraense Emilio Goeldi (MPEG); Collection of Mammals Museum of the Federal University of Mato Grosso (CMUFMT); Museum of Zoology, Federal University of Western Pará (MZUFOPA); Institute of Scientific and Technological Research of the State of Amapá (IEPA). (DOCX 22 kb) [file 12862_2019_1421_MOESM1_ESM.docx]

Additional file 1 - Table of specimens analyzed. Species, Number of individuals/sex, locality, state of origin (SO), diploid number (2n), fundamental number (FN) and deposit of specimen vouchers analyzed in the present study. Museu Paraense Emilio Goeldi (MPEG); Collection of Mammals Museum of the Federal University of Mato Grosso (CMUFMT); Museum of Zoology, Federal University of Western Pará (MZUFOPA); Institute of Scientific and Technological Research of the State of Amapá (IEPA).

| Species | Individuals/Sex | Locality | SO | 2n | FN | Voucher |
| --- | --- | --- | --- | --- | --- | --- |
| *Lampronycteris brachyotis* | 1. F | Faro town | PA | 32 | 60 | MPEG |
|  | 1. F | Alta Floresta | MT | 32 | 60 | CMUFMT |
| *Micronycteris Megalotis* | 1. M | Caixuanã National Forest | PA | 40 | 68 | MPEG |
|  | 1. F | Zoo at Faculdades Integradas  do Tapajós | PA | 40 | 68 | MZUFOPA |
|  | 1. M | Loure­nço town | AP | 40 | 68 | IEPA |
|  | 1. M | Laranjal do Jari town | AP | 42 | 70 | IEPA |
|  | 1. F (4) M | Urucará town | AM | 40 | 68 | MPEG |
|  | 1. M | Jacareacanga town | PA | 40 | 68 | MPEG |
| *Micronycteris microtis* | 1. M | Urucará town | AM | 40 | 68 | MPEG |
|  | 1. M | Floresta Nacional do Amapá | AP | 40 | 68 | IEPA |
| *Micronycteris homezi* | 1. M | Santa Bárbara do Pará town | PA | 28 | 52 | MPEG |
| *Micronycteris minuta* | 1. F (1) M | Santa Bárbara do Pará town | PA | 28 | 52 | MPEG |
|  | 1. F | Belém city | PA | 28 | 52 | MPEG |
|  | (5) F (3) M | Mexiana Island | PA | 28 | 52 | MPEG |
|  | 1. M | Carajás National Forest | PA | 28 | 52 | MPEG |
|  | 1. M | Laranjal do Jari town | AP | 28 | 52 | IEPA |
|  | 1. F (1) M | Urucará town | AM | 28 | 52 | MPEG |
|  | 1. F | Santarém city | PA | 28 | 52 | MZUFOPA |
|  | 1. M | Cotriguaçú town | MT | 28 | 52 | CMUFMT |
